# The feasibility of a dose painting procedure to treat prostate cancer based on mpMR images and hierarchical clustering

**DOI:** 10.1186/s13014-021-01906-2

**Published:** 2021-09-20

**Authors:** Seyed Masoud Rezaeijo, Bijan Hashemi, Bahram Mofid, Mohsen Bakhshandeh, Arash Mahdavi, Mohammad Saber Hashemi

**Affiliations:** 1 Department of Medical Physics, Faculty of Medical Sciences, Tarbiat Modares University, Al-Ahmad and Chamran Cross, 1411713116 Tehran, Iran; 2 Department of Radiation Oncology, Faculty of Medicine, Shahid Beheshti University of Medical Sciences, Tehran, Iran; 3 Department of Radiology Technology, Faculty of Paramedical Sciences, Shahid Beheshti University of Medical Sciences, Tehran, Iran; 4 Department of Radiology, Modares Hospital, Shahid Beheshti University of Medical Sciences, Tehran, Iran; 5 Department of Aerospace Engineering, Iowa State University, Ames, IA 50011 USA

**Keywords:** Prostate cancer, Dose painting, Hierarchical clustering, mpMR images, IMRT, 3DCRT

## Abstract

**Background:**

We aimed to assess the feasibility of a dose painting (DP) procedure, known as simultaneous integrated boost intensity modulated radiation Therapy (SIB-IMRT), for treating prostate cancer with dominant intraprostatic lesions (DILs) based on multi-parametric magnetic resonance (mpMR) images and hierarchical clustering with a machine learning technique.

**Methods:**

The mpMR images of 120 patients were used to create hierarchical clustering and draw a dendrogram. Three clusters were selected for performing agglomerative clustering. Then, the DIL acquired from the mpMR images of 20 patients were categorized into three groups to have them treated with a DP procedure being composed of three planning target volumes (PTVs) determined as PTV1, PTV2, and PTV3 in treatment plans. The DP procedure was carried out on the patients wherein a total dose of 80, 85 and 91 Gy were delivered to the PTV1, PTV2, and PTV3, respectively. Dosimetric and radiobiologic parameters [Tumor Control Probability (TCP) and Normal Tissue Complication Probability (NTCP)] of the DP procedure were compared with those of the conventional IMRT and Three-Dimensional Conformal Radiation Therapy (3DCRT) procedures carried out on another group of 20 patients. A post-treatment follow-up was also made four months after the radiotherapy procedures.

**Results:**

All the dosimetric variables and the NTCPs of the organs at risks (OARs) revealed no significant difference between the DP and IMRT procedures. Regarding the TCP of three investigated PTVs, significant differences were observed between the DP versus IMRT and also DP versus 3DCRT procedures. At post-treatment follow-up, the DIL volumes and apparent diffusion coefficient (ADC) values in the DP group differed significantly (*p*-value < 0.001) from those of the IMRT. However, the whole prostate ADC and prostate-specific antigen (PSA) indicated no significant difference (*p*-value > 0.05) between the DP versus IMRT.

**Conclusions:**

The results of this comprehensive clinical trial illustrated the feasibility of our DP procedure for treating prostate cancer based on mpMR images validated with acquired patients’ dosimetric and radiobiologic assessment and their follow-ups. This study confirms significant potential of the proposed DP procedure as a promising treatment planning to achieve effective dose escalation and treatment for prostate cancer.

*Trial registration*: IRCT20181006041257N1; Iranian Registry of Clinical Trials, Registered: 23 October 2019, https://en.irct.ir/trial/34305.

## Background

Prostate cancer is not uniformly distributed in the prostate and may involve several areas of the prostate (so-called multifocal) [1]. External beam radiotherapy (EBRT) is one of the standard techniques used for treating these tumors [2, 3]. Treatment procedures like intensity-modulated radiotherapy (IMRT) and volumetric modulated arc therapy (VMAT) are known as high flexible EBRT methods for delivering dose prescription [4, 5]. Satisfactory results have been reported for treating low-risk tumors with these procedures with common prescribed doses [6–11]. However, histopathological assessment of prostatectomy specimens reveals intraprostatic lesions (IPLs) also referred to as dominant intraprostatic lesions (DILs). One of the recognized leading causes of prostate cancer recurrence and consequently radiotherapy failure are the DILs [11, 12]. Hence, controlling and treating these tumors can be improved by increasing the prescribed dose. In an overall dose-escalation procedure [13–15], a uniform high level dose distribution is used instead of a non-uniform dose distribution known as dose painting (DP) procedure [16–18]. The DP is known as a simultaneous integrated boost-IMRT (SIB-IMRT), which offers the opportunity to treat both whole prostate volume and DIL volume simultaneously at different doses. The SIB-IMRT or DP uses only one radiation treatment plan during the entire course of treatment.

In DP procedure, based on the features extracted from functional images, the prostate DILs can be defined for delivering a non-uniform dose boost to improve tumor control without increasing clinical complications [13, 14, 19–23]. To properly control the tumor and prevent more treatment-related toxicity, it is recommended that DILs be identified with multi-parametric MRI (mpMR images) including: T2 weighted (T2W), diffusion-weighted MRI (DW-MRI), and dynamic contrast-enhanced MRI (DCE-MRI) sequences. However, the DIL contouring is a manual procedure wherein a radiologist and radiotherapist decide on the DIL areas to have them included in the target based on medical data and mpMR images. For example, an apparent diffusion coefficient (ADC) map and a volume transfer constant (Ktrans) derived from DW-MRI and DCE-MR images have been investigated extensively as prognostic and predictive biomarkers in a wide variety of tumors [24]. With such imaging modalities used in this study to distinguish the tumors from normal tissues, the reported sensitivity levels have ranged from 54–84 to 59–87%, while the specificity levels have ranged from 74–100 to 74–84% for the DW-MRI (ADC) and DCE-MRI, respectively. It has also been shown that the use of mpMR images can increase the accuracy of diagnosing prostate cancer and reduce the number of patients who will require repeated biopsies [25]. However, mpMR images still renders some limitations. Variability is reported regarding diagnostic accuracy and inter-reader agreement, being generally dependent on reader experience. Therefore, for DIL classification, a problem arises on how to profit from these imaging modalities.

Machine learning algorithms are artificial intelligence techniques that adapt statistical and probabilistic tools to learn from preceding examples and then predict new trends. Application of machine learning in medical imaging aims to assist the specialist in diagnosing diseases. Computer-aided diagnosis is one of the first applications of these new algorithms, which incorporates machine learning classifiers trained to distinguish lesions from normal tissue. In image elaboration, machine learning algorithms can directly learn the structure labeling of each image voxel to segment DILs [26]. Machine learning techniques have already been applied for detecting prostate cancer [26–28]. On the other hand, hierarchical clustering is an exploratory statistical method used for identifying groups based on similarity between the acquired data [28]. Hierarchical clustering outcomes can be interpreted since its’ algorithm is clear, and the relationship between the input and output can be visualized in a dendrogram. This clustering has been extensively studied in many fields, such as functional MRI in connectivity analysis [29]. Nevertheless, hierarchical clustering is infrequently applied to mpMR images. Despite the advantages of dose escalation to DILs, the DP method has not yet been generally introduced as a standard method for prostate radiotherapy due to the escalating dose and its’ potential complications. Hence, it appears that multidisciplinary and comprehensive research is needed to utilize clinically DP method and eliminate existing doubts. According to our knowledge, there is no report on the feasibility of DP procedure to treat DILs based on mpMR images and hierarchical clustering with machine learning. Therefore, in this study hierarchical clustering was used for the classification of DILs in prostate cancer patients. Then, the DP planning was performed on a group of patients undergoing prostate radiotherapy based on classification of their DILs based on mpMR images and hierarchical clustering with a machine learning technique. Finally, to investigate the feasibility of DP procedure, the dosimetric and radiobiologic parameters of the DP treatment plans were extracted and compared with those of another group of 20 patients treated with the conventional IMRT. In addition, for the patients treated with the IMRT, a 3-dimensional conformal radiation therapy (3DCRT) planning was investigated. Besides, a post-treatment follow-up was performed four months post-radiotherapy for the two group of the patients to assess their tumor response to the DP and IMRT treatment procedures.

## Materials and methods

**Fig. 1:**
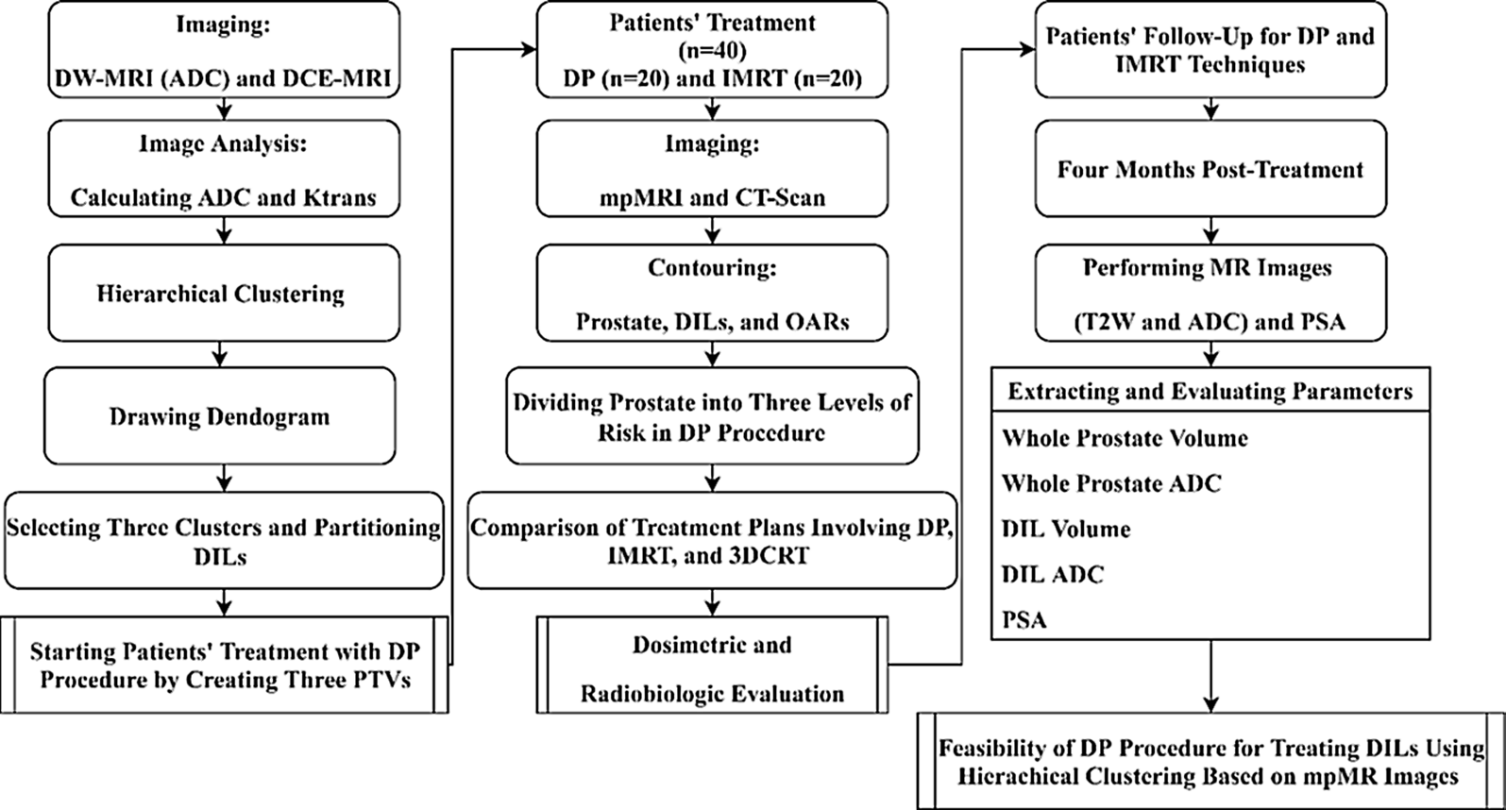
The general framework of the study design

Our "institutional ethics committee" approved this randomized clinical trial study that was also registered by our national registry of clinical trials. All the procedures carried out in this research were in accordance with the Helsinki Declaration (1964) and its’ amendments. Informed consent was obtained for any experimentation with the subjects. The general framework of the study is depicted as a diagram in Fig. 1.

### Patients' characteristics for designing hierarchical clustering

The patients with the following inclusion criteria were regarded for training hierarchical clustering: biopsy-proven prostate cancer with localized intermediate or high-risk disease and no evidence of metastatic disease. The exclusion criteria were: previous prostate radiotherapy, prostatectomy, and contraindications to MRI including, cardiac pacemakers, prosthetic valves, and metal implants. Considering these criteria, the mpMR images of 120 patients, namely the ADC and Ktrans, were used to create hierarchical clustering. Characteristics of the patients are presented in Table 1.

**Table 1:** Characteristics of the patients

Parameters	Value^a,b^
Age (years)	68 ± 7
Initial prostate-specific antigen (iPSA) level (ng/ml)	25 ± 11
Gleason score 7	38
Gleason score 8 or 9	82
DIL volume (cm^3^)	1.9 ± 1.39
DIL ADC (10^−3^ mm^2^/s)	1.1 ± 0.39
DIL Ktrans (min^−1^)	2.1 ± 1.35

^a^Mean and standard deviation (Mean ± SD) values are reported for age, PSA, DIL volume, DIL ADC, and DIL Ktrans

^b^The values stated for the Gleason score indicate the number of patients with the relevant scores

### MR Imaging

The mpMR images were obtained from April 2019 to February 2020 by a 1.5 T MR system (Siemens Medical Solutions, Germany) consisting of T2W, DW-MRI, and DCE-MRI. The MR imaging parameters are summarized in Table 2 [30].

**Table 2:** MRI parameters

Sequence	TR/TE (ms)	Slice thickness (mm)	Matrix size	FOV (mm)	Voxel size (mm)
T2W-axial	7920/93	3	320 × 320	200	0.62 × 0.62 × 3
T2W-coronal	7570/101	3	256 × 256	200	0.78 × 0.78 × 3
T2W-sagittal	7680/101	3	256 × 256	200	0.78 × 0.78 × 3
DW-MRI	4600/72	3	112 × 112	199	1.78 × 1.78 × 3
DCE-MRI	4.5/1.69	3	192 × 192	259	1.35 × 1.35 × 3

TR/TE: Repetition time/echo time; FOV: field of view

For DW-MRI, three different b-values were used. An ADC map was automatically calculated. using the following formula:

1 $${\text{SI}}_{{\text{i}}} = {\text{SI}}_{0} \times {\text{e}}^{{( - {\text{bi}} \times {\text{ADC}})}}$$

where SI_i_ is the signal intensity measured on the ith b-value image, bi the corresponding b-value (being: 50, 800, 1200 s/mm^2^), and SI_0_ a variable estimating the exact signal intensity for b = 0 s/mm^2^. The DCE-MRI was performed by administrating 15 mL of gadolinium at a rate of 2 mL/s as an intravenous bolus injection. It should be noted that the Ktrans maps were generated by fitting a Tofts model for each voxel and all the patients using an individual-based arterial input function (AIF). The Fire Voxel version 324B software package (New York University, USA) was used to display and analyze the DCE-MRI and ADC images.

### Delineation of the DILs

All the MR images were reviewed by a radiologist (AM, with 11 years of experience in interpreting prostate MRI). For each case, a combined review of axial T2W, ADC, and DCE images was performed. The DILs were manually segmented by drawing regions of interest (ROIs) along the visible tumor margins in each slice of the ADC maps, with a careful reference to the biopsy-proven regions. The ROI-based mean ADC (10^−3^ mm^2^/s) was calculated by averaging the ADC values for the entire slices wherein the DILs were visible. The same radiologist manually drew an ROI on the right external iliac artery of the DCE images for every patient. The AIF was obtained from post-averaging of the ROI at each time point. Subsequently, the DILs were segmented manually based on the corresponding ADC map by drawing an ROI around the enhanced DILs in each slice. Then, the standard Tofts model was applied to generate the Ktrans.

### Hierarchical clustering for mpMR images

**Fig. 2:**
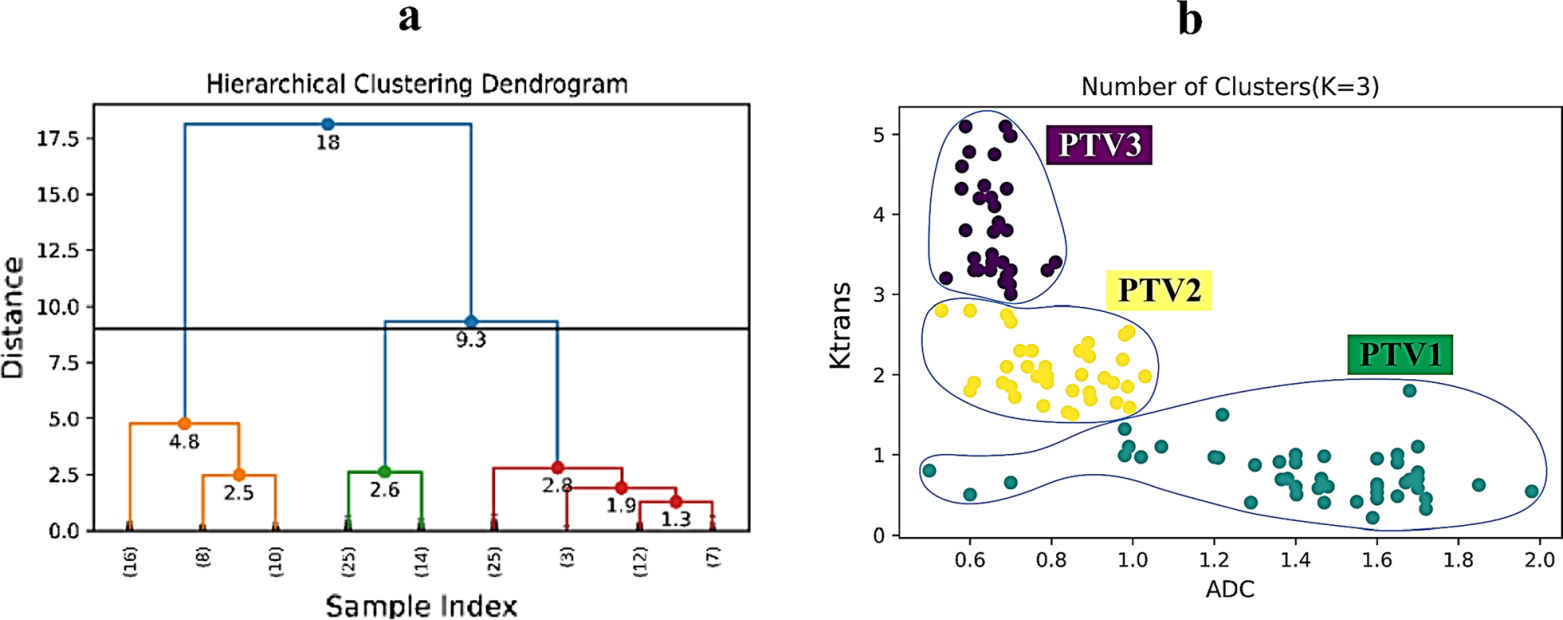
**a** Dendrogram (the **“distance”** indicates the closeness of either individual data points or clusters and the **“sample index”** is the number of data); **b** clustering classes based on the opinion of the radiologist and oncologist in which PTV3 correlates with ADC < 0.8 mm^2^/s and Ktrans > 3; PTV2 with 0.65 < ADC ≤ 1 mm^2^/s and 1 < Ktrans ≤ 3; and finally PTV1 with ﻿1 < ADC mm^2^/s and Ktrans < 1

After calculating the ADC and Ktrans, the DILs were divided into different levels of risk. Hence, for categorizing the DILs, the hierarchical clustering was used. Categorizing data into clusters is the primary purpose of clustering algorithms, such that similar objects are grouped in the same cluster according to specific metrics. Defining the optimal number of clusters in a data set is critical in partitioning clustering, such as agglomerative clustering, which requires a user to specify the number of clusters, k, to be created. There is no precise response to this question. The optimal number of clusters is anyhow subjective and depends on the technique applied for estimating similarities and the parameters used for partitioning. A famous and straightforward solution involves investigating the dendrogram produced using hierarchical clustering to see if it suggests an appropriate number of clusters. Hierarchical clustering methods start from many clusters that are objects. The objects are joined gradually into the clusters, up to the final cluster obtained from all the objects. In each stage, one or two objects and one or two clusters are merged. The hierarchy is an outcome of the fact that larger clusters are regularly obtained by merging smaller ones. Thus, hierarchical clustering is used to draw the typical result of the dendrogram. A dendrogram is a visualization in the form of a tree showing the order and distances of merges during the hierarchical clustering. However, the challenging problem is that there is no golden method to pick optimal clusters. Therefore, if we want to argue for a certain number of clusters, what we are interested in is a considerable jump in the dendrogram's distance that would be typical. In general, this can be done simply by counting the number of intersections with vertical lines of the dendrogram to get the number of formed clusters based on the chosen cut-off value of maximum distance (Fig. 2a). A cut-off value of nine was selected in our study, as the jump was pretty obvious. With horizontal cut at different levels in the dendrogram, it was seen that three clusters are a good selection for clustering (Fig. 2a). In Fig. 2a, the y-axis (distance) measures the closeness of either individual data points or clusters, and the x-axis (sample index) is the number of data. Therefore, we selected three clusters for performing agglomerative clustering (Fig. 2b). It should be noted that the ward linkage method was used to draw the dendrogram and clustering. Besides, the Euclidean was applied for the distance metrics. After determining the number of clusters by drawing the dendrogram (K = 3), partitioning clustering was performed. It is also necessary to mention that the ADC and Ktrans values needing neither feature selection nor pre-processing methods were applied as the input to the hierarchical clustering. As shown in Fig. 2b, the DILs were partitioned into three groups, and the patients' treatment with the DP procedure was started by creating three levels of risk or planning target volume, including: PTV1, PTV2, and PTV3. The PTV3 and PTV1 were regarded as the high-risk and low-risk prostate regions, respectively, while regions with an intermediate DIL probability as the PTV2. Finally, the radiologist and oncologist concluded that PTV3 correlates with ADC < 0.8 mm^2^/s and Ktrans > 3. In addition, PTV2 correlated with 0.65 < ADC ≤ 1 mm^2^/s and 1 < Ktrans ≤ 3 and also PTV1 with 1 < ADC mm^2^/s and﻿ Ktrans < 1. In summary, classifying the DILs for delivering different levels of treatment doses in the DP procedure was provided by our in-house clustering algorithms as described above.

## Treatment of patients

### Patients’ characteristics

**Fig. 3:**
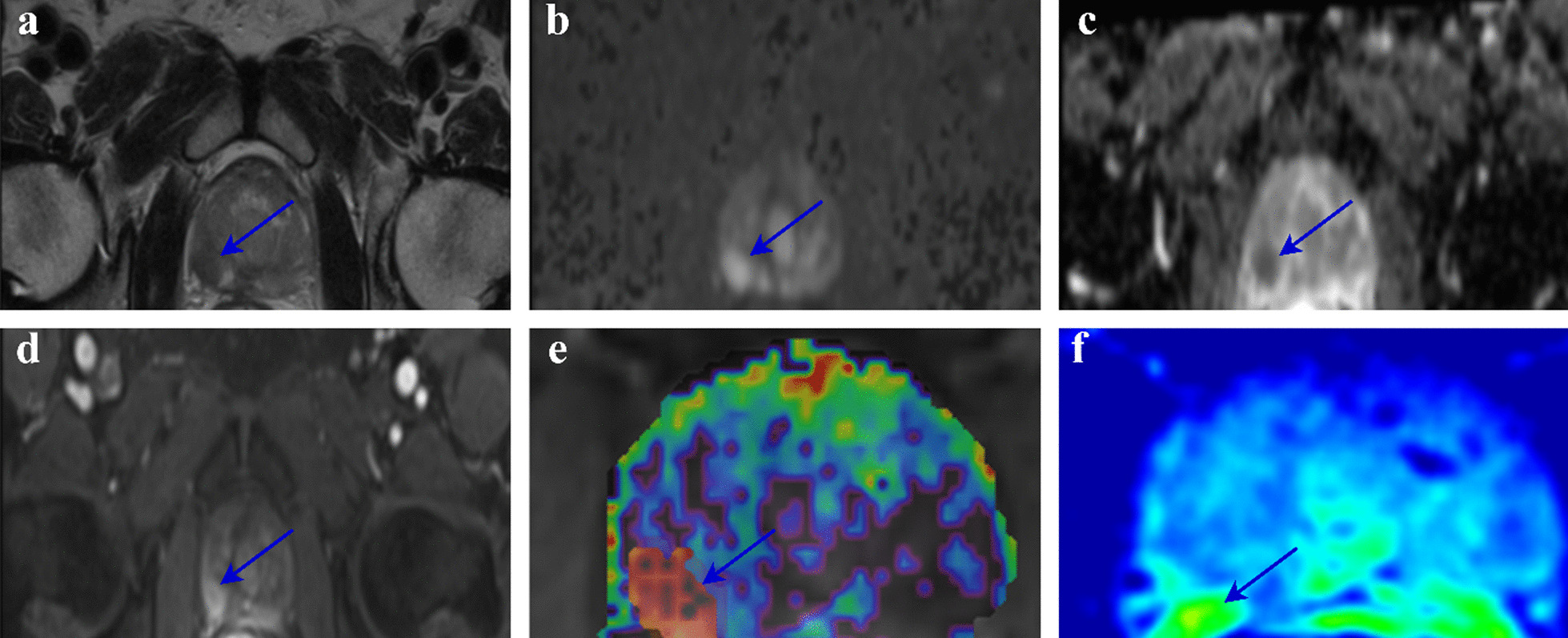
The mpMR images of a 68-year-old man with a DIL in the right lobe (arrows). DIL volume: 0.6 cm^3^, whole prostate volume: 42.3 cm^3^, PSA level: 38 ng/mL, Gleason score: 3 + 4. The DIL is clearly visible as a hypointense lesion on T2W (**a**), a hyperintense lesion on DWI (**b**), a hypointense lesion on ADC map (**c**), an early enhancing lesion on the DCE-MRI (**d**), Ktrans map (**e**), and the reconstructed map (**f**)

The current study is a randomized clinical trial. An independent trial center performed the randomization process. If the patients met the inclusion criteria and provided informed consent, the physician contacted the trial center. The inclusion and exclusion criteria were regarded as similar to those described before for training hierarchical clustering. Furthermore, the patients diagnosed with prostate cancer were underwent androgen-deprivation therapy (ADT) within six months of diagnosis. The treatment was carried out on two groups of patients, including 20 patients treated with the DP procedure and 20 other patients with the conventional IMRT procedure. The patients' characteristics are listed in Table 3. The mpMR images data is displayed for one of the patients in Fig. 3, including the T2W, DWI-MRI, ADC map, DCE-MRI, Ktrans, and reconstructed map. Therefore, based on the ADC and Ktrans parameters, the DILs were classified and their dose boost were defined. For all the patients treated with the IMRT procedure, an alternative 3DCRT planning was also investigated. It must be noted that the tumor response to the treatment or the DIL’s disappearance was considered as the endpoint of this clinical trial study.

**Table 3:** Characteristics of the patients

Parameters	Value ^a,b^	
	DP procedure	IMRT procedure
Age (years)	71 ± 8	73 ± 9
Initial PSA level (ng/ml)	24.86 ± 9.5	22.22 ± 10.8
Gleason score		
7	9	8
8–10	11	12
Number of lesions	34	37
Whole prostate volume (cm^3^)	37.18 ± 9.17	35.66 ± 10.56
DIL volume (cm^3^)	1.73 ± 1.41	1.67 ± 1.45
Whole prostate ADC (10^−6^ mm^2^/s)	1122 ± 142	1154 ± 124.4
DIL ADC (10^−6^ mm^2^/s)	894.9 ± 253	884.3 ± 242
DIL Ktrans (min^−1^)	3.6 ± 2.3	3.1 ± 1.9

^a^Mean and standard deviation (Mean ± SD) values are reported for age, PSA, Whole prostate volume, DIL volume, whole prostate ADC, DIL ADC, and DIL Ktrans

^b^The values stated for the Gleason score are the number of patients with the mentioned scores

### Patients’ contouring

**Fig. 4:**
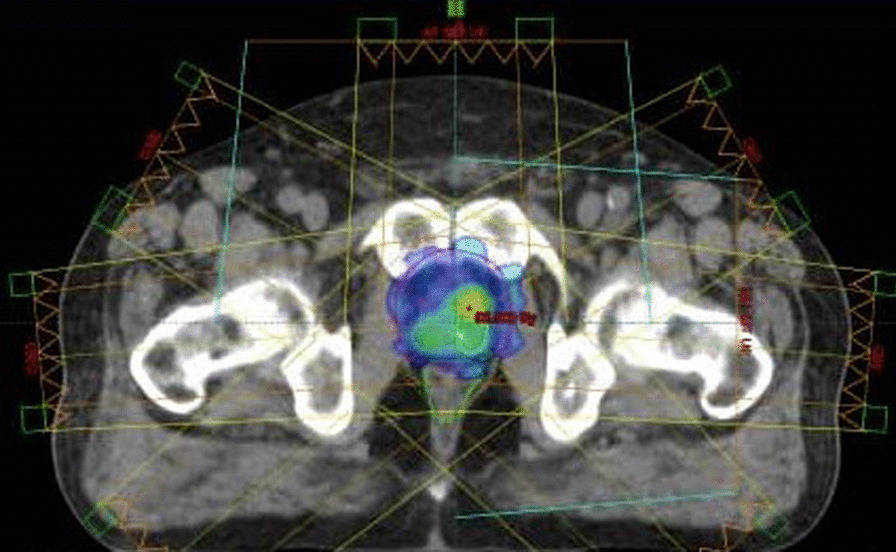
The distributions of the prescripted dose for a patient treated with the DP procedure

All the patients' images, including mpMR images and CT scan, were registered using the rigid registration technique. Before scanning and treatment sessions, all the patients were instructed to empty their bowels and drink 300 ml of water for 20 min. It is obvious that if possible, the patients should ideally maintain a full bladder, but the bladder volume in patients with prostate cancer is reported to be unknown. Bladder volume reproducibility is typically enhanced through water consumption, but the optimal amount of water to be consumed remains unclear [31, 32]. When the rectal filling differed considerably on the patients' CT-scan and mpMR images, new scans were performed to minimize the fusion uncertainty between the imaging modalities. The planning target volumes (PTVs) and DILs were delineated on the patients' CT scans and mpMR images based on the European Society of Radiation & Oncology and Advisory Committee on Radiation Oncology Practice (ESTRO-ACROP) guideline [33]. The DILs, prostate, and rectum were contoured, as shown in Fig. 4. According to the hierarchical clustering, the DILs classification was performed by creating three levels of risk (PTVs).

### Treatment planning procedure

For both of the DP and IMRT procedures, all the patients were treated by applying seven radiation fields at various gantry angles of 0°, 65°, 95°, 135°, 225°, 265° and 295° using a 6 MV photon beam. Three PTVs were created in the DP procedure, including PTV1 as a DIL with ADC > 1 mm^2^/s and Ktrans < 1, PTV2 as a DIL with 0.65 < ADC ﻿< 1 mm^2^/s and 1 < Ktrans < 3, and PTV3 as a DIL with ADC < 0.8 mm^2^/s and Ktrans > 3. It should be noted that no boost dose was considered for the PTV1 which was contoured similar to the IMRT and 3DCRT procedures. Hence, for the PTV1, 6 mm in the posterior and 7 mm in the anterior, cranial-caudal, and transverse margins of the prostate were considered. Nevertheless, to consider any inaccuracies in the DIL delineation, a 5 mm margin was added to the DIL volumes for the PTV2 and PTV3 [15, 16, 34]. A total dose of 80 Gy (2 Gy/fraction), 85 Gy (2.125 Gy/fraction), and 91 Gy (2.275 Gy/fraction) with a SIB technique was delivered to the PTV1, PTV2, and PTV3, respectively. Implementing the DP procedure was impossible for five patients because their prescribed dose led to rectum's overdosage. These patients were excluded from the group of 20 patients who underwent the DP procedure. For the IMRT and 3DCRT techniques, the relevant margins chosen for the prostate were 6 mm along the posterior, and 7 mm along the cranial-caudal, transverse, and anterior directions. In addition, a 10 mm margin was used for seminal vesicles. For both the IMRT and 3DCRT procedures, a total dose of 80 Gy (2 Gy/fraction) to the whole PTV was planned. For the 3DCRT procedure similar to the IMRT and DP procedures, the seven-field technique was applied using the same 6 MV photon beam.

### Dosimetric and radiobiologic evaluation

The plans were evaluated using the dose-volume histograms (DVHs) derived from isodose distributions. Based on the DVHs and the dose constraint presented in Table 4, relevant dosimetric variables were calculated and reported for the PTVs and organs at risk (OARs) volumes.

**Table 4:** The dose constraints used for dosimetric evaluation of the radiotherapy procedures [35]

OARs	Dose-volume parameter (%)
Bladder^a^	V80 < 15
	V75 < 25
	V70 < 35
	V65 < 50
Rectum^a^	V75 < 15
	V70 < 20
	V65 < 25
	V60 < 35
	V50 < 50
Femoral heads^b^	V40 < 40^c^
	V50 < 10

^a^QUANTEC recommendations

^b^RTOG recommendations

^c^V40, structure volume receiving at least 40 Gy and son on for other dose-volume parameters

For biological evaluation of the plans, BioSuite (Updated 10–01–2018) software was used [36]. The Normal Tissue Complication Probability (NTCP) was estimated by using the relative seriality model [37]. This model describes the response of an organ with a mixture of serial and parallel structure. The following equation gives the NTCP:

1 $$NTCP = \left\{ {1 - \mathop \prod \limits_{I} \left[ {1 - P(D_{i} )^{s} } \right]^{{v_{i} }} } \right\}^{{{\raise0.7ex\hbox{$1$} \!\mathord{\left/ {\vphantom {1 s}}\right.\kern-\nulldelimiterspace} \!\lower0.7ex\hbox{$s$}}}}$$

where vi is the fractional organ volume receiving a dose Di and P (Di) is the complication probability. Relevant parameters used in the model for bladder, rectum, and femoral heads are shown in Table 5 in which the 50% response dose is named as D50, γ as the maximum normalized value of the dose–response gradient, and s describes the relative contribution of each type of architecture which is equal to unity for a fully serial and zero for a fully parallel organ. In addition, the α/β ratio is a measure of the fractionation sensitivity of the cells and the organ-specific dose. The Tumor Control Probability (TCP) was calculated for three PTV using the LQ-Poisson “Marsden” TCP model [38] with the following parameters: α = 0.155 Gy^−1^, α/β = 1.5 Gy, α spread = 0.058 Gy^−1^, and clonogenic density = 10^7^ cm^−3^.

**Table 5:** The relevant relative seriality model parameters used for the NTCP calculations [39]

OARs	D50 (Gy)	γ	s	α/β
Bladder	69.56	1.7	0.35	3
Rectum	69.75	2.3	0.84	3
Femoral heads	65	2.7	1	3

### Patients’ follow-up

MRI and PSA follow-up were performed four months after the radiotherapy for all the 40 patients (20 patients treated with the DP and another 20 patients with the IMRT). All of the patients were examined with T2W and DW-MRI (ADC). The whole prostate volume, DIL volume, ADC value of the whole prostate, ADC value of the DIL, and PSA were assessed and then compared with their counterpart pre-treatment values. Post-radiotherapy, to calculate the ADC of the patients with no residual DILs, the ROI was drawn in the same area as used initially in the pre-treatment.

### Statistical analysis

Data was analyzed using the GraphPad Prism software (GraphPad, USA). D’Agostino test was used to assess the normality of data. One-way ANOVA and Kruskal–Wallis statistical methods were applied for multiple comparisons. The confidence interval (CI) and mean rank were used as statistically significant indexes for one-way ANOVA and Kruskal–Wallis statistical methods. For analyzing and comparing the follow-up data of the pre- and post-treatment, the paired *t*-test was used. An independent *t*-test was also used to compare the response to the treatment resulted from the DP and IMRT procedures. *P*-values less than 0.05 were considered statistically significant for paired *t*-test and independent *t*-test. The attributed number to the pre-treatment group was chosen 100. Then, the whole prostate volume, DIL volume, ADC value of the whole prostate, ADC value of the DIL, and PSA for the post-treatment groups were computed as the percentages of it. The vertical bars in histograms represent the standard deviation (SD) of the means.

## Results

### Dosimetric and radiobiologic analysis

**Fig. 5:**
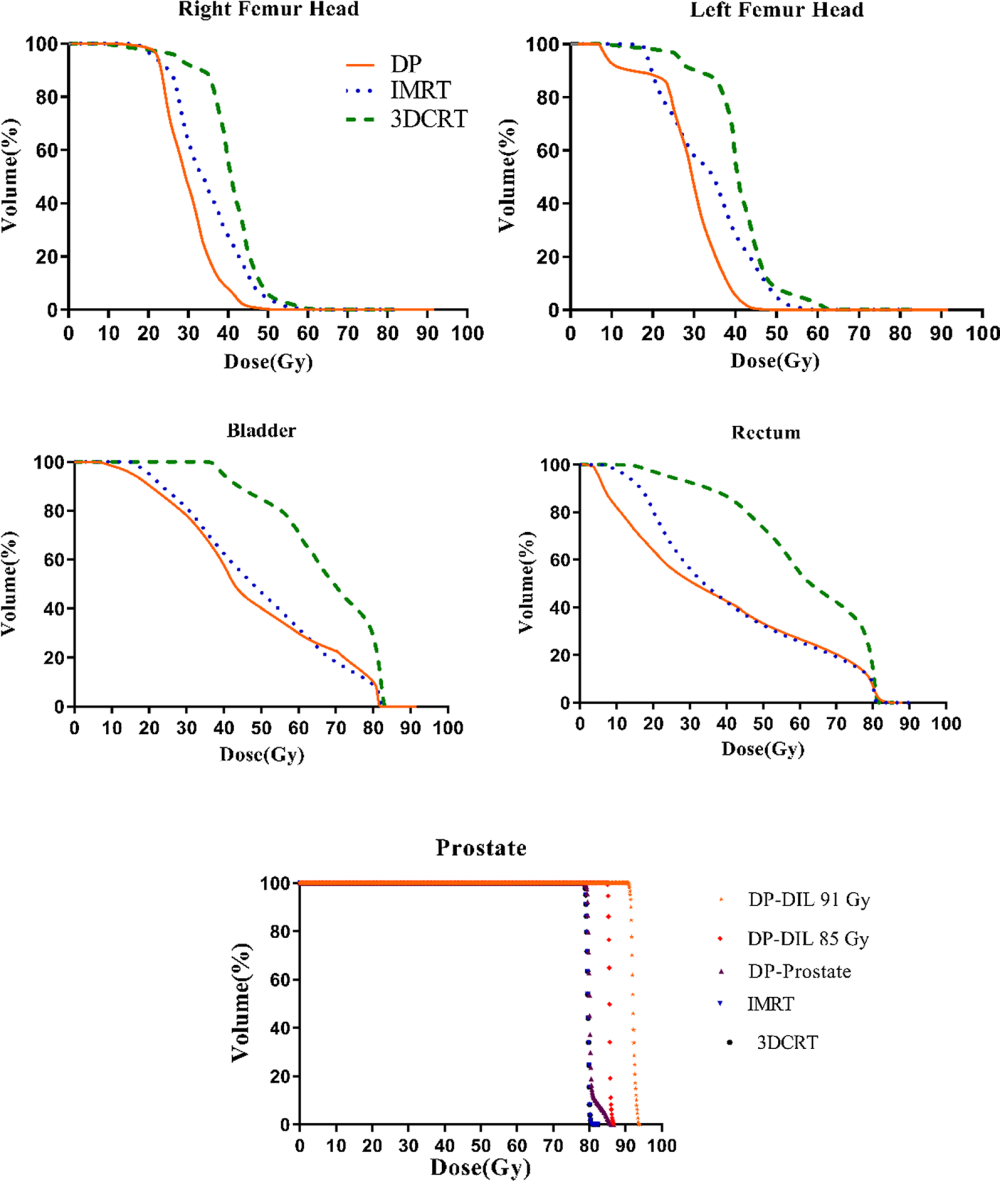
DVHs of the DP, IMRT, and 3DCRT procedures for the prostate and OARs

Table 6 shows the dosimetric variables of the OARs for the DP, conventional IMRT, and 3DCRT procedures. The differences of the OARs’ doses among the DP, IMRT, and 3DCRT procedures were significant. For all the dosimetric variables, no significant difference was found between the DP and IMRT procedures. For the bladder, the mean of dosimetric variables for the DP was slightly higher than the IMRT. But for the rectum, the mean of the dosimetric variables was lower for the DP procedure. Figure 5 shows the cumulative dose-volume histograms (DVH) of the prostate and OARs for the DP, IMRT, and 3DCRT procedures. As shown, the bladder, rectum, and femoral heads dosimetric parameters are improved with the DP procedure compared with other procedures.

**Table 6:** Comparison of dosimetric variables in three techniques of DP, IMRT, and 3DCRT

Structure	Dosimetric variable	DP	IMRT	3DCRT	95% CI of difference		
		Mean ± SD	Mean ± SD	Mean ± SD	DP versus IMRT	DP versus 3DCRT	IMRT versus 3DCRT
Bladder	V80 (%)	3.83 ± 2.9	3.34 ± 2.02	14.16 ± 8.33	− 3.555 to 4.551	− 14.38 to − 6.273*	− 14.88 to − 6.771*
	V75 (%)	14.02 ± 3.13	13.87 ± 6.67	22.31 ± 9.24	− 3.109 to 5.678	− 11.44 to − 2.654*	− 12.73 to − 3.939*
	V70 (%)	19.2 ± 5.1	18.59 ± 8.88	26.91 ± 11.48	− 5.492 to 7.861	− 13.72 to − 0.3676*	− 14.90 to − 1.552*
	V65 (%)	22.99 ± 9.9	21.85 ± 11.19	29.57 ± 13.37	− 8.702 to 7.900	− 15.33 to 1.272	− 14.93 to 1.673
	Mean dose (Gy)	37.5 ± 5.66	35.15 ± 10.14	44.9 ± 9.23	− 2.762 to 10.07	− 13.64 to − 0.8027*	− 17.29 to − 4.458*
Rectum	V75 (%)	11.6 ± 2.2	12.85 ± 2.13	34.85 ± 3.41	− 3.389 to 0.7814	− 25.43 to − 21.26*	− 24.13 to − 19.96*
	V70 (%)	17.05 ± 2.65	17.23 ± 4.91	42.12 ± 4.63	− 5.255 to 1.508	− 29.44 to − 22.68*	− 27.56 to − 20.80*
	V65 (%)	19.22 ± 2.52	20.08 ± 2.36	48.11 ± 6.77	− 4.247 to 2.121	− 33.17 to − 26.80*	− 32.11 to − 25.74*
	V60 (%)	25.28 ± 3.38	25.43 ± 3.59	57.21 ± 6	− 3.609 to 3.329	− 35.40 to − 28.46*	− 35.26 to − 28.32*
	V50 (%)	32.27 ± 4.5	35.64 ± 3.78	70.12 ± 6	− 8.324 to 0.2627	− 42.18 to − 33.59*	− 38.27 to − 29.43*
	Mean dose (Gy)	39.7 ± 3.12	41.45 ± 2.12	57.25 ± 3.98	− 4.659 to 0.5579	− 20.52 to − 15.31*	− 18.55 to − 13.18*
Right femur head	V40 (%)	8.64 ± 3.98	12.89 ± 6	44.75 ± 10.96	− 9.475 to 1.131	− 41.21 to − 30.60*	− 37.03 to − 26.43*
	V50 (%)	0.63 ± 0.73	1.1 ± 0.82	21.8 ± 4.54	− 3.053 to 0.8153	− 22.95 to − 19.08*	− 21.83 to − 17.96*
	Mean dose (Gy)	28.42 ± 2.57	29.03 ± 3.21	39.86 ± 4.85	− 1.678 to 3.192	− 13.54 to − 8.668*	− 14.30 to − 9.425*
Left femur head	V40 (%)	9.05 ± 3.09	12.46 ± 6.47	45.55 ± 11	− 8.272 to 2.000	− 40.02 to -29.74*	− 36.88 to − 26.61*
	V50 (%)	0.75 ± 0.9	1.89 ± 1.36	22.98 ± 3.77	− 2.778 to 1.509	− 23.76 to -19.47*	− 23.12 to − 18.84*
	Mean dose (Gy)	29.01 ± 1.31	29.79 ± 2.02	40.17 ± 4.6	− 1.760 to 3.422	− 13.10 to − 7.920*	− 13.93 to − 8.751*

^*^Significant difference

Although there was no significant difference between the DP and IMRT for femoral heads, the mean variables were lower for the DP procedure. As shown in Table 7, no significant difference was observed in the values of NTCP for the OARs between the DP and IMRT procedures. Similar to the dosimetric variables for the bladder, the NTCP of the OARs for the DP was more than IMRT, while it was less for the rectum. There was a significant difference in the OARs’ NTCP values between the IMRT vs. 3DCRT and the DP vs. 3DCRT. As shown in Table 7, for the TCP in three investigated PTVs, significant differences are observed between the DP versus IMRT and DP versus 3DCRT.

**Table 7:** Comparison of TCP and NTCP variables in three techniques of DP, IMRT, and 3DCRT

Radiobiologic variable	Structure	DP	IMRT	3DCRT	95% CI of difference/ Mean Rank		
		Mean ± SD	Mean ± SD	Mean ± SD	DP versus IMRT	DP versus 3DCRT	IMRT versus 3DCRT
TCP (%)	PTV1	72.39 ± 1.57	70.52 ± 1.47	70.17 ± 1.99	0.5828 to 3.162*	0.9328 to 3.512*	− 0.9397 to 1.640
	PTV2	89.9 ± 1.2	70.52 ± 1.47**	70.17 ± 1.99**	18.24 to 20.52*`	18.59 to 20.87*	− 0.8446 to 1.545
	PTV3	91.86 ± 1.02	70.52 ± 1.47**	70.17 ± 1.99**	19.80 to 22.87*	20.15 to 23.22*	− 0.9044 to 1.604
NTCP (%)	Bladder	5.4 ± 2.1	4.8 ± 2	13.31 ± 4.4	− 1.867 to 3.027	− 10.35 to − 5.460*	− 11.07 to − 5.908*
	Rectum	16.54 ± 2.9	19.2 ± 3.7	43.27 ± 4.4	− 5.575 to 0.1254	− 29.58 to − 23.88*	− 26.86 to − 21.15*
	Right femur head	0.0002 ± 0.0004	0.005 ± 0.01	1.87 ± 0.98	− 2.56	− 23.31*	− 20.75*
	Left femur head	0.0001 ± 0.0003	0.003 ± 0.01	1.7 ± 0.46	− 1.5	− 24.74*	− 23.25*

^*^Significant difference

^**^PTV2 and PTV3 were not considered for the IMRT and 3DCRT procedures. Hence, PTV1 values were considered as PTV2 and PTV3 values in IMRT and 3DCRT procedures

### Patients’ follow-up

**Fig. 6:**
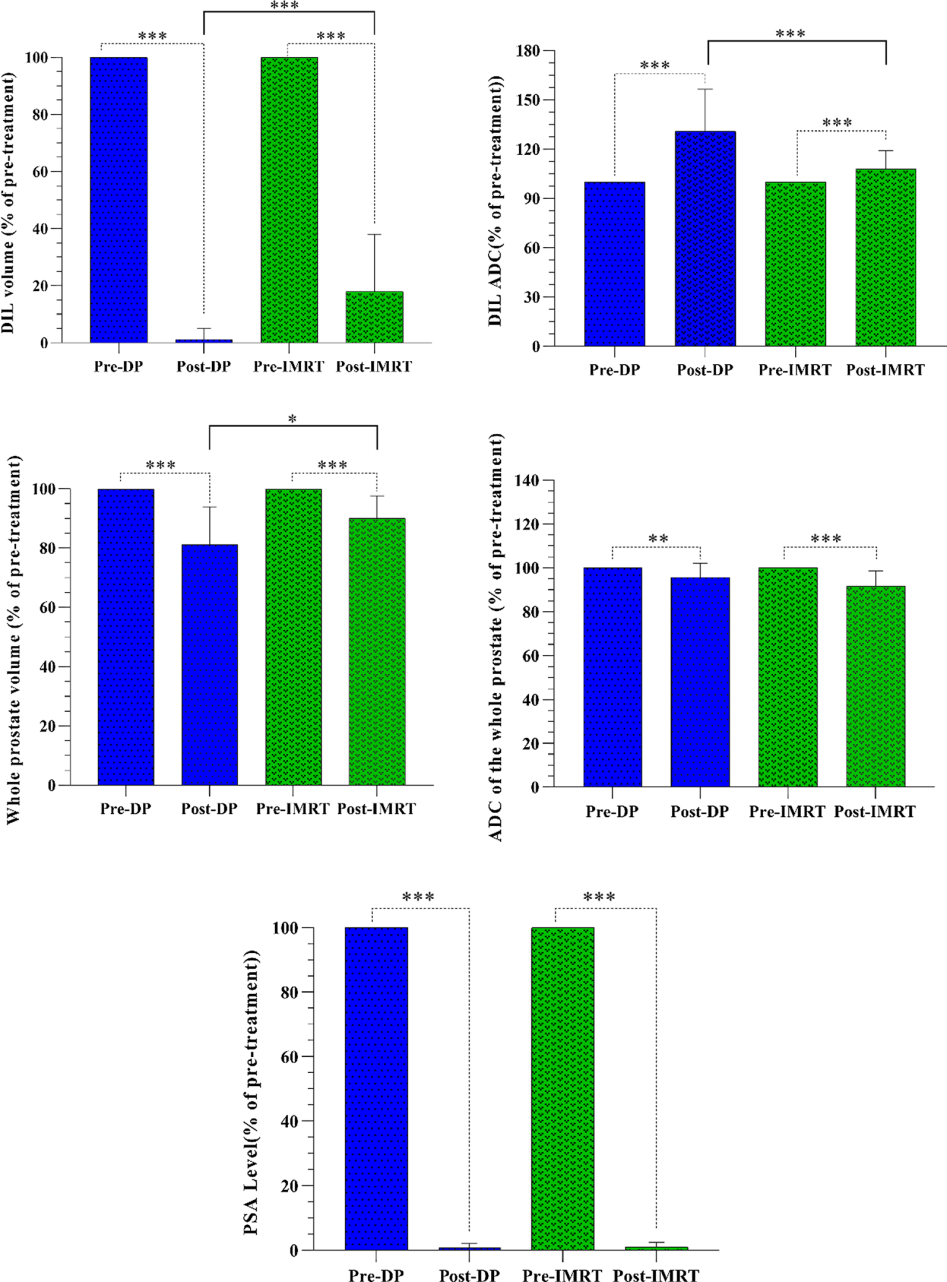
The DIL volume, ADC value of the DIL, whole prostate volume, ADC value of the whole prostate, and PSA for both of the pre- and post-radiotherapy groups. The attributed number to the pre-treatment group was chosen 100. Then, the whole prostate volume, DIL volume, ADC value of the whole prostate, ADC value of the DIL, and PSA for the post-treatment groups were computed as percentages of it. Vertical bars represent standard deviation (SD) of the mean. *p < 0.05, **p < 0.01, ***p < 0.001

**Fig. 7:**
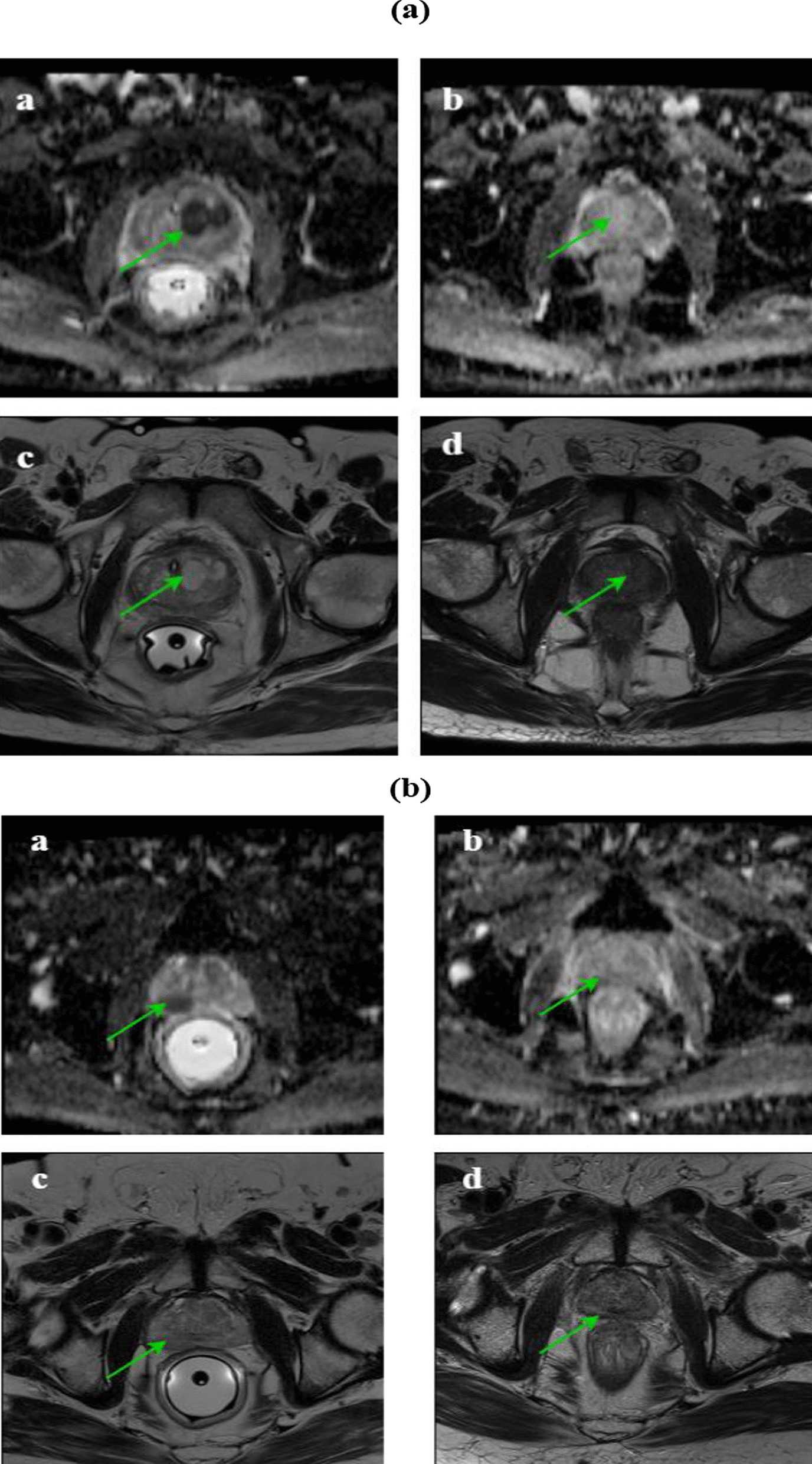

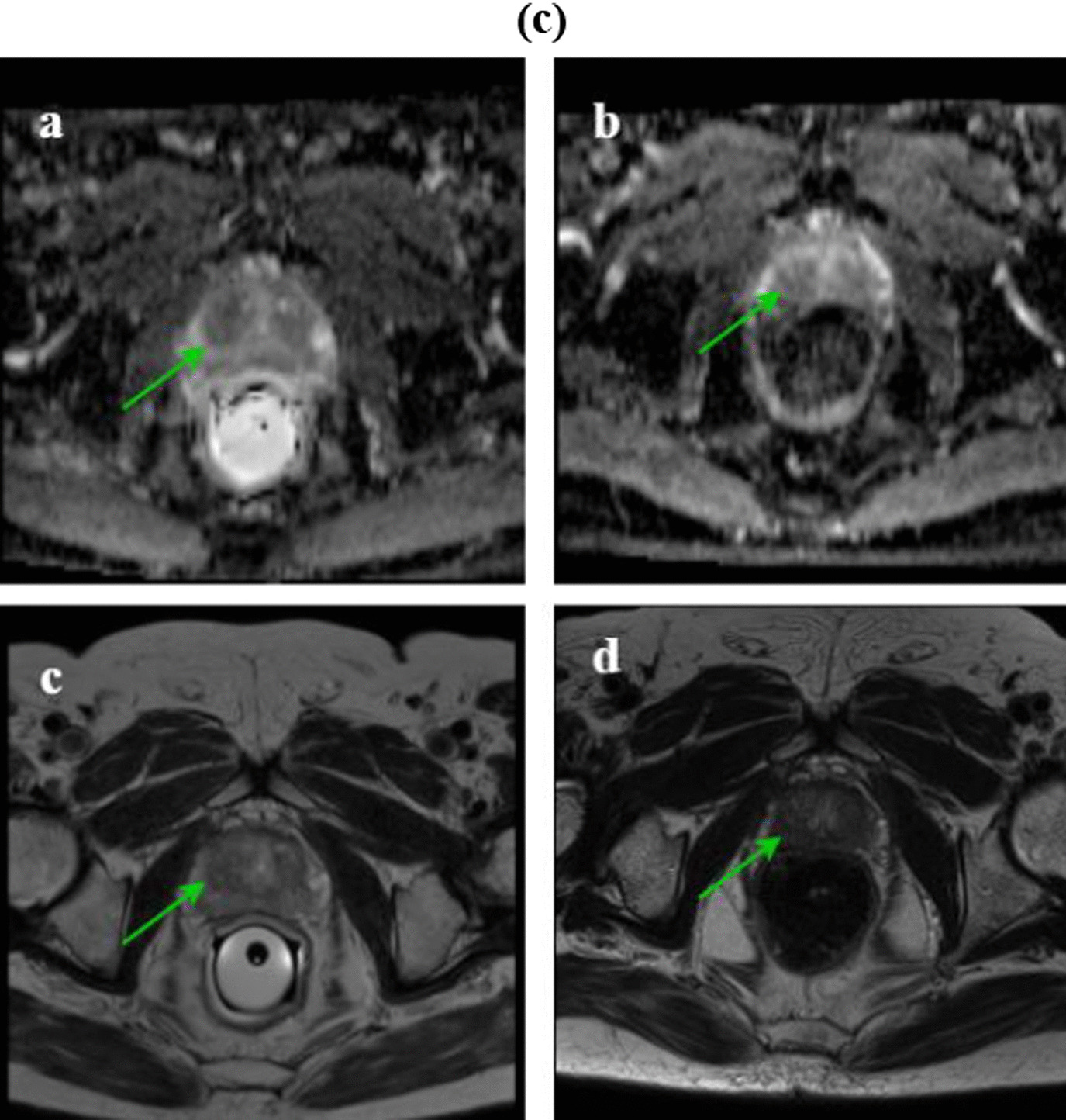
**a** The MR images of a 67-year-old man. Pre-radiotherapy, the axial T2W image shows a hypointense DIL in the right apex (**c**) and the DIL volume was 4.8 cm^3^ in the ADC image and the ADC value was 572 × 10^−6^ mm^2^/s (**a**). Post-radiotherapy, the axial T2W image shows the diffusely intermediate signal of the right apex (**d**) and the DIL volume was not detected in the ADC image, and the ADC value was 1210 × 10^−6^ mm^2^/s. These finding indicate a good response to the treatment (**b**). **b** The MR images of a 77-year-old man. Pre-radiotherapy, the axial T2W image shows a hypointense DIL in the right midgland (**c**) and the DIL volume was 4.6 cm^3^ in the ADC image and the ADC value was 820 × 10^−6^ mm^2^/s (**a**). Post-radiotherapy, the axial T2W image shows a diffusely intermediate signal of the right midgland (**d**) and the DIL volume was 0.38 cm^3^ in ADC image, and the ADC value was 920 × 10^−6^ mm^2^/s. These findings indicate a poor response to the treatment (**b**).** c** The MR images of a 64-year-old man. Pre-radiotherapy, the axial T2W image shows a hypointense DIL in the left peripheral zone (**c**) and the DIL volume was 1.2 cm^3^ in the ADC image and the ADC value was 651 × 10^−6^ mm^2^/s (**a**). Post-radiotherapy, the axial T2W image shows the diffusely intermediate signal of the left midgland (**d**). Post-radiotherapy, the DIL volume was 0.72 cm^3^ in ADC image and the ADC value was 736 × 10^−6^ mm^2^/s. These findings indicate a poor response to the treatment (**b**)

The DIL volume and ADC value for both pre- and post-treatment groups were significantly different (*p*-value < 0.001). Post-radiotherapy, the DIL volumes and ADC values in the DP group significantly differed from IMRT (*p*-value < 0.001) as presented in Table 8 and Fig. 6. Pre-treatment, 34 DILs were diagnosed in the DP group from which 30 DILs were treated entirely post-treatment (Fig. 7a) and 4 DILs were not completely disappeared (Fig. 7b). However, the number of DILs post-treatment in the DP was less compared with IMRT group. The percentage change of the ADC of the DILs post-treatment of the DP was greater than IMRT. Pre-treatment, 37 DILs were identified in the IMRT group. Although a decrease in the volume of DILs was observed post-treatment, the volume of DILs was quite apparent for 26 cases (Fig. 7c) in this group. This finding suggests a poor response to the IMRT treatment. There was a significant difference for all of the parameters pre- and post-treatment with either the DP or IMRT including: the whole prostate volume (*p*-value < 0.001), whole prostate ADC (with a *p*-value of 0.008 and < 0.001 for the DP procedure and IMRT procedures, respectively), and PSA (*p*-value < 0.001) for both groups (Fig. 6). However, post-treatment, comparing these parameters with the DP and IMRT indicated no significant difference between them with a *p*-value of 0.06, and 0.64 for the whole prostate ADC and PSA, respectively, while a significant difference was observed for the whole prostate volume (*p*-value = 0.01).

**Table 8:** The follow-up results

Parameters	Mean (range)					
	DP procedure			IMRT Procedure		
	Pre-radiotherapy	Post-radiotherapy	Percentage change	Pre-radiotherapy	Post-radiotherapy	Percentage change
DIL volume (cm^3^)	1.73 (0.1–4.9)	0.037 (0–0.4)	98.83 (78.9–100)	1.67 (0.04–5.1)	0.24 (0–1.2)	81.95 (38.89–100)
Whole prostate volume (cm^3^)	37.18 (21–56)	30.28 (18–46.9)	18.16 (4.16–51.35)	35.66 (20.77–62.37)	32.41 (18–53)	10.11 (2.4–26.32)
DIL ADC (10^−6^ mm^2^/s)	894.9 (572–1420)	1123 (823–1500)	32.18 (0.84–111.5)	884.3 (580–1462)	939.2 (698–1400)	10.35 (0.08–47.1)
Whole prostate ADC (10^−6^ mm^2^/s)	1122 (869–1389)	1072 (856–1325)	6.81 (1.44–15.85)	1154 (976–1421)	1059 (723–1312)	8.45 (1.41–20)
PSA level (ng/ml)	24.86 (13.3–45)	0.17 (.001–0.98)	99.2 (95–99.9)	22.22 (5.2–48)	0.15 (.002–0.8)	99 (93.4–100)

## Discussion

To improve the validity of mpMR image-based for prostate tumor classifications, we first developed a hierarchical clustering. With horizontal cut at different levels in the dendrogram, it was seen that three clusters are a good selection for clustering. Hence, three PTVs were created and used for the DP procedure. Akamine et al. [40] investigated whether hierarchical clustering can differentiate prostate cancer and normal tissue by using mpMR images. In their study, hierarchical clustering was constructed using mpMR images, including diffusion kurtosis imaging and dynamic contrast-enhanced MRI from 40 tumor and normal tissues in the peripheral zone and 23 tumor and normal tissues in the transition zone. They concluded that hierarchical clustering could accurately differentiate prostate cancer and normal tissue. Similarly, our results showed that ADC images and Ktrans extracted from the DWI and DCE-MRI images could be used to treat prostate cancer using the DP procedure. Furthermore, the feasibility of the DP procedure based on mpMR images as an alternative technique for IMRT and 3DCRT in prostate cancer radiotherapy was investigated and analyzed in our study. The treatment planning was compared according to the dosimetric and radiobiological parameters, including the TCP and NTCP. In the DP group, in addition to the prostate, the TCP was calculated for the DILs for α/β of 1.5 Gy. Radiobiological parameters were calculated using the LQ Poisson “Marsden” TCP model [38] and relative seriality model.

The feasibility of the DP procedure has also been investigated clinically by Lips et al. [20]. Based on their mpMR images and drawing two PTVs, a total dose of 77 Gy in 2.2 Gy/fraction has been prescribed to the whole prostate. A dose of 95 Gy in 2.7 Gy/fraction has been delivered to the DILs with a margin of 4 mm, announcing that it is possible to deliver a dose escalation to the DILs without compromising the dose constraints for the rectum and bladder. The main difference between our study and Lips et al. is the prescribed dose to the whole prostate and DILs based on the quantitative parameters of mpMR images, patient follow-up, and analysis of imaging parameters for the two DP and IMRT groups. However, despite different dose escalation and drawing of three PTVs in our study, we did not exceed the dose limits of the OARs, including bladder and rectum. As shown, the feasibility of dose escalation is possible for DILs based on mpMR images. Kerkmeijer et al. [16] investigated whether using DP procedure with EBRT for the visible macroscopic tumor increases biochemical disease-free survival (bDFS) for 571 patients with localized prostate cancer. In their study, a total dose of 95 Gy (2.7 Gy/fraction) with a SIB technique was delivered to the DILs on mpMR images. They concluded that while simultaneously sparing OARs, focal therapy in prostate cancer improves the tumor control. Similarly, our results showed the significant potential of the DP procedure in our study as a more promising treatment planning for an effective and complication-free dose escalation in prostate cancer. It must be noted that the specific DP treatment planning settings designed and implemented in our study (such as: prescribed dose and PTVs, evaluation metrics, and imaging parameters for the DP and IMRT groups) differed with those used previously in the above study. In addition, in our study the DP planning was performed based on the classification of DILs according to the mpMR images and hierarchical clustering. Uzan et al. [23] planned 11 patients with a DP procedure based on mpMR images using radiobiological evaluation, including NTCP-bleeding, NTCP-incontinence, and TCP. They claimed that the DP procedure has the ability to achieve significant escalation of the DIL dose. With α/β of 3 Gy, their mean TCP-boost increased from 71% for standard IMRT plans to 83.1% for DP plans, whereas NTCP did not exceed 6.2%. However, in our study, the mean TCP-prostate increased from 70.52% and 70.17% for IMRT and 3DCRT procedures to 72.39% for DP procedures. However, the mean TCP-DIL for PTV2 and PTV3 vs. IMRT increased by 89.9% and 91.86%, respectively. Therefore, for the TCP-prostate and TCP-DIL, there was a significant difference between the DP vs. IMRT and DP vs. 3DCRT. Due to the relative seriality model vs. the LKB model, the NTCP for the rectum did exceed 6.2%. Of note, we performed a radiobiological evaluation vs. the number of therapy sessions by using Biosuite software. To have the same radiobiological effect of the DP procedure, we need to increase the number of treatment sessions and the dose delivery for the IMRT and 3DCRT procedures. Gronlund et al. [19] mapped the ADC data to the Gleason score using probability distributions. They claimed that the DP prescriptions increase the TCP without increasing dose burdens for the OARs. In Housri et al. study [41], MR images of the prostate were evaluated for the DILs assessment. A total dose of 75.6 Gy (1.8 Gy/fraction) was prescribed to the whole prostate, and a dose of 151.2 Gy (200% of the prescribed dose) in 3.6 Gy/fraction was delivered to the DILs. According to their study, the distance between the lesion and the rectum restricted the ability to plan high-dose radiation to DILs. Moreover, they announced that DIL planning seems possible to treat DILs. In our study, the distance between the lesion and rectum was one of the limiting factors of the prescribed dose. Consequently, for five patients, implementing the DP procedure was not possible because the prescribed doses lead to an overdosage of the rectum. These patients were excluded from the DP treatment. However, generally it was possible to deliver a dose escalation to the DILs without compromising the dose constraints for the rectum.

As reported, the DIL volumes pre- and post-treatment were different in both groups. In the DP group, the ratio of therapeutic volumes was much lower than in the IMRT group, and for most DILs, the therapeutic volume was disappeared post-treatment. It can also be said that ADC is one of the essential parameters for examining the response to the post-radiotherapy treatment. Hence, increasing the ADC post-treatment can be considered a good criterion of DIL response to treatment. However, some of the lesions are resistant to high-dose treatment; therefore, the primary treatment regimen cannot completely eradicate the DILs. Furthermore, the volume and ADC of the whole prostate and PSA were measured pre- and post-treatment, and it was found that these parameters were different in the IMRT and DP groups. However, no significant difference was detected between the two groups for the whole prostate ADC and PSA.

Due to the limitation enforced by our "Organizational Ethics Committee" to select the minimum required sample size in our clinical trials, we were unable to include more patients. Therefore, future extra studies with a large sample size are suggested, if approved by relevant official ethics committees. Furthermore, in our clinical trial, only two hierarchical clustering input parameters of DWI were used to build the model, and other quantitative parameters like radiomic features were not included. Since additional radionics features as hierarchical clustering input may improve the accuracy of differentiating the DILs, the optimization of hierarchical clustering input parameters is recommended to be assessed to build more accurate hierarchical clustering models in future studies.

## Conclusion

We performed a comprehensive clinical trial study and illustrated the feasibility of the DP procedure to treat prostate cancer based on mpMR images validated with the patients' dosimetric and radiobiologic parameters and their follow-ups. Our study confirms significant potential of the proposed DP procedure as a more promising treatment planning for an effective and complication free dose escalation in prostate cancer.

## Data Availability

The datasets used and/or analyzed during the current study are available from the corresponding author on reasonable request only for personal uses.

## Abbreviations

EBRT: External beam radiotherapy
IMRT: Intensity-modulated radiotherapy
VMAT: Volumetric modulated arc therapy
OARs: Organs at risk
IPL: Intraprostatic lesion
DIL: Dominant intraprostatic lesion
DP: Dose painting
mpMR images: Multi-parametric MRI
T2W: T2 weighted
DW-MRI: Diffusion-weighted MRI
DCE-MRI: Dynamic contrast-enhanced MRI
ADC: Apparent diffusion coefficient
Ktrans: Volume transfer constant
AIF: Arterial input function
ROIs: Regions of interest
PTVs: Planning target volumes
SIB: Simultaneous integrated boost
DVHs: Dose-volume histograms
CI: Confidence interval
